# Impact of the COVID-19 pandemic on Muslim older immigrants in Edmonton, Alberta: A community-based participatory research project with a local mosque

**DOI:** 10.17269/s41997-023-00764-7

**Published:** 2023-03-30

**Authors:** Amyna Ismail Rehmani, Khadija Abdi, Esra Ben Mabrouk, Tianqi Zhao, Bukola O. Salami, Allyson Jones, Hongmei Tong, Jordana Salma

**Affiliations:** 1 Faculty of Nursing, University of Alberta, Edmonton, AB Canada; 2 School of Public Health, University of Alberta, Edmonton, AB Canada; 3 Faculty of Rehabilitation Medicine, University of Alberta, Edmonton, AB Canada; 4 Robbins Health Learning Centre, MacEwan University, Edmonton, AB Canada

**Keywords:** COVID-19 pandemic, Immigrants, Older adults, Community-based participatory research, Pandémie de COVID-19, immigrants, personnes âgées, recherche participative communautaire

## Abstract

**Objective:**

Older Muslim immigrants experience multiple vulnerabilities living in Canada. This study explores the experiences of Muslim older adults during the COVID-19 pandemic to identify ways to build community resilience as part of a community-based participatory research partnership with a mosque in Edmonton, Alberta.

**Methods:**

Using a mixed-methods approach, check-in surveys (*n* = 88) followed by semi-structured interviews (*n* = 16) were conducted to assess the impact of COVID-19 on older adults from the mosque congregation. Quantitative findings were reported through descriptive statistics, and thematic analysis guided the identification of key findings from the interviews using the socio-ecological model.

**Results:**

Three major themes were identified in consultation with a Muslim community advisory committee: (a) triple jeopardy leading to loneliness, (b) decreased access to resources for connectivity, and (c) organizational struggles to provide support during the pandemic. The findings from the survey and interviews highlight various supports that were missing during the pandemic for this population.

**Conclusion:**

The COVID-19 pandemic exacerbated the challenges associated with aging in the Muslim population and contributed to further marginalization, with mosques being sites of support during times of crises. Policymakers and service providers must explore ways of engaging mosque-based support systems in meeting the needs of older Muslim adults during pandemics.

**Supplementary Information:**

The online version contains supplementary material available at 10.17269/s41997-023-00764-7.

## Résumé

**Objectif:**

Les immigrants musulmans âgés vivant au Canada sont confrontés à des vulnérabilités multiples. Cette étude explore les expériences des personnes âgées musulmanes durant la pandémie de COVID-19 afin d’identifier des moyens pour renforcer la résilience communautaire dans le cadre d’un partenariat de recherche participative communautaire (CBPR) avec une mosquée à Edmonton, en Alberta.

**Méthodes:**

À l’aide d’une approche à méthodes mixtes, des enquêtes de contrôle (n = 88) suivies d’entretiens semi-dirigés (n = 16) ont été menés pour évaluer l’impact du COVID-19 sur les personnes âgées de la congrégation de la mosquée. Les résultats quantitatifs ont été rapportés au moyen de statistiques descriptives, tandis que l’analyse thématique a guidé l’identification des principaux résultats des entretiens à l’aide du modèle socio-écologique.

**Résultats:**

Trois thèmes majeurs ont été identifiés en concertation avec un comité consultatif de la communauté musulmane : a) triple péril menant à la solitude, b) accès réduit aux ressources pour la connectivité, et c) luttes organisationnelles pour fournir un soutien pendant la pandémie. Les résultats de l’enquête et des entretiens mettent en évidence le manque d’aides diverses à cette population durant la pandémie.

**Conclusion:**

La pandémie de COVID-19 a exacerbé les défis associés au vieillissement de cette population et a contribué à une marginalisation supplémentaire, les mosquées étant des sites de soutien en temps de crise. Les décideurs politiques et les prestataires de services doivent explorer les moyens d’engager les systèmes d’aide basés dans les mosquées pour répondre aux besoins des adultes musulmans âgés pendant les pandémies.

## Introduction

According to the recent Canadian census, almost one in four people is a landed immigrant in Canada (Statistics Canada, 2022). Alberta is home to over 100,000 Muslims, the majority of whom are first- and second-generation immigrants (Statistics Canada, 2017). Muslim older adults experience similar challenges to other older immigrant groups, including social isolation, loneliness, and lack of access to aging-supportive resources (Salma & Salami, 2020). Identity politics, racism, and Islamophobia can further marginalize older immigrants from mainstream resources and services (Reitz et al., 2017). Furthermore, immigrants and refugees are more likely to be concerned about the economic and social impacts of the pandemic than the general population (Johnson et al., 2021). Pandemic conditions exacerbated the vulnerabilities of many immigrants because of language barriers that limited access to timely information, socio-economic constraints that impacted access to the necessities of daily life, and barriers to health care access and utilization (Đoàn et al., 2021); however, the specific impact on older immigrants has not been extensively explored.

Several provincially and federally funded services provide social and economic support to Canadian older adults, but these services cannot meet all needs of immigrants, including many older Muslims (Salma & Salami, 2020). Local religious and ethnocultural community organizations help to meet the common needs of Canadian immigrant communities and support spiritual, mental, and social well-being (Salami et al., 2019). No studies have explored the COVID-19 pandemic experiences of older Muslims or factors to mitigate their vulnerability during a pandemic. This study used an existing academic-mosque partnership (McCabe et al., 2014) to achieve the overall objective of improving the well-being of Muslim immigrant older adults, and this paper focuses on the experiences and needs of this population.

## Methods

### Theoretical framework

**Fig. 1:**
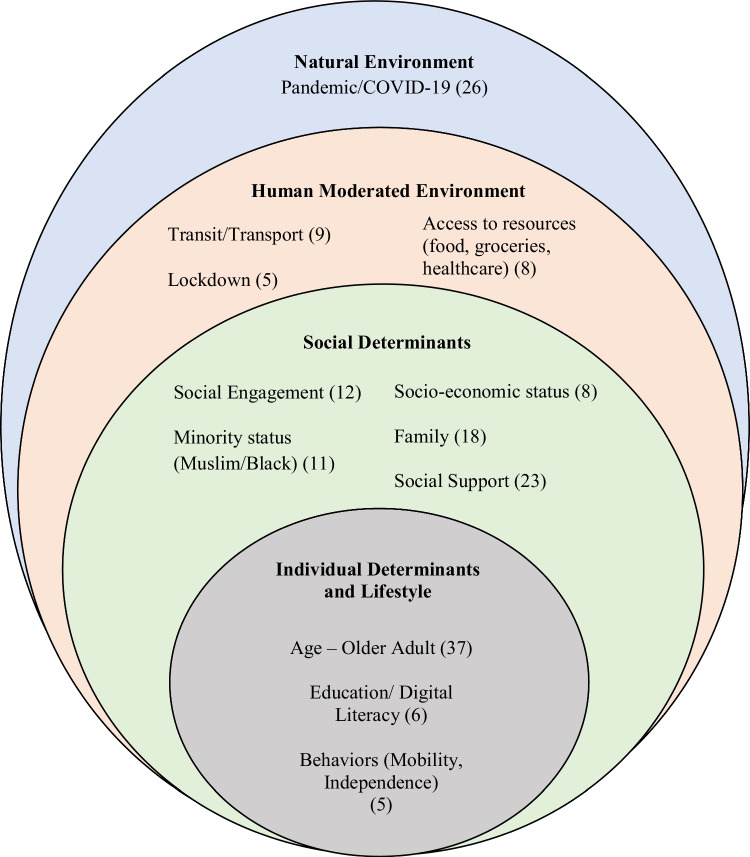
Number of pandemic-related references to dimensions of the socio-ecological model. Adapted from “System models for resilience in gerontology: Application to the COVID-19 pandemic” (Klasa et al., 2021)

A socio-ecological model (Fig. 1) was used as a sensitizing framework during data analysis and included five domains: individual lifestyle, individual determinants, social determinants, human-moderated environment, and natural environment (Klasa et al., 2021). The model conceptualized the factors at the intrapersonal, interpersonal, and community levels that impacted the well-being of Muslim immigrant older adults (Ellison et al., 2021).

### Study design

The study was part of a larger community-based participatory research (CBPR) project with a local mosque in Edmonton, Alberta, and used mixed methods and rapid qualitative designs (Vindrola & Johnson, 2020). A community advisory group of 10 Muslim women from diverse ethnocultural communities informed the objectives and activities of the research project. Three main goals were identified: (a) to explore the experiences of Muslim older adults during the COVID-19 pandemic; (b) to identify gaps in meeting their needs; and (c) to implement feasible and mosque-driven intervention(s) to address these gaps. We report on the first two objectives in this paper.

### Recruitment and study sample

A community liaison recruited participants through convenience sampling by calling the phone numbers of older adults who attended religious and social programs at the mosque, and through an information booth at the mosque during Friday prayers. If participants provided initial consent after being introduced to the study, they were screened for study inclusion. Inclusion criteria were self-identifying as Muslim and being 50 years of age or older. Older age can be perceived differently across immigrant communities; hence, we utilized the lower age cut-off of 50 years. Being an immigrant was not a direct inclusion criterion, considering the majority of Muslims in Canada are first- or second-generation immigrants (Statistics Canada, 2017).

Stakeholders with a working knowledge of Muslim communities and who were part of the mosque’s network were recruited for qualitative interviews to add insight into the findings from older adults. Stakeholders were recruited by phone or email using contact information provided by the main community partner mosque.

### Data collection

Data collection occurred from April 2021 to October 2021, coinciding with the third and fourth waves of the pandemic in Canada (Detsky & Bogoch, 2021). Ethics approval was obtained from the University of Alberta Research Ethics Board (Pro00109010).

#### Quantitative survey

The mosque leadership requested a quick approach to “check in” with older adults in their congregation. A 10–15-min survey was designed using input from the research team with expertise in aging and from mosque leadership. The survey was piloted by the community liaison with five participants from the mosque and then modified to resolve unclear wording or response choices. The survey consisted of 24 multiple-choice questions regarding sociodemographic profiles, health status, and participants’ views on mosque-based programs. Research assistants contacted the interested participants by phone and obtained verbal or written consent after a detailed explanation of the study and prior to data collection. Verbal consent was more feasible when targeting older adults with lower digital literacy. The 24-item survey was administered by the research assistants (fluent in English, Arabic, Urdu, Somali) over the phone in each participant’s preferred language. After survey completion, participants were invited to participate in an in-depth qualitative interview to expand on their experiences.

#### Qualitative interviews

Qualitative interviews were essential for understanding the needs of this population and for tailoring subsequent mosque interventions. The surveys informed the focus of the interviews (see interview guide in supplementary file), which consisted of questions reflecting the general experience of living through the pandemic, challenges faced, strategies utilized for coping, and the need for support (e.g., social, emotional) during the pandemic. Stakeholders were asked about the situation of Muslim older adults in their communities and any barriers or challenges they encountered when responding to the needs of older adults. Participants provided written or verbal consent before the interview. The primary author with expertise in qualitative methods and an in-depth knowledge of the Muslim community conducted the semi-structured narrative interviews online via Zoom or over the phone, depending on participants’ preference. The primary author identifies as a Muslim woman and has been working with the community since 2015. The interviews were audio-recorded and lasted 30–60 min. This small sample size was deemed sufficient using the principles of “information power” because of the narrow aim of the study and the specificity of the sample (older Muslims from one mosque congregation) (Malterud et al., 2016).

### Data analysis

#### Quantitative analysis

Quantitative findings from the survey were reported through descriptive statistics, and summary statistics were performed using Excel and Stata. Age was dichotomized to determine any differences with characteristics.

#### Qualitative analysis

Qualitative interviews were thematically analyzed (Braun & Clarke, 2006) with NVivo 12 software. Audio-recordings were reviewed by two independent researchers to extract key codes and related quotes in a template format. Findings were compared and discussed in team meetings. This approach of data analysis has acceptable reliability when conducted by experienced researchers (Vindrola & Johnson, 2020). The advisory committee was included in the analysis via a presentation to share preliminary findings and elicit feedback on how these findings reflected the experiences of their communities. Team members (AR and JS) involved in the data analysis belonged to the Edmonton Muslim community and had established relationships of trust with the mosque. Hence, knowledge of the social, cultural, and political context helped contextualize the qualitative data.

## Results

### Participant characteristics

A total of 88 people participated in the check-in survey, and 10 of these agreed to complete qualitative interviews. Those who refused to complete surveys or interviews after being introduced to the study cited lack of time or interest. The survey respondents comprised 46 (52.2%) women and 42 (47.7%) men with a mean age of 63 (SD: 9.6) years; most respondents were young-old with a small sample older than 76 years. Participants were mostly immigrants from diverse countries of origin who had lived in Canada for over 10 years. The majority lived with spouses, children, or both, with a small number of respondents living alone. Education levels were mostly secondary and post-secondary and sources of income were variable. The qualitative-interview participants from this sample were five women and five men from a variety of language and cultural groups (see Table 1). Six stakeholders were also recruited for interviews by the community liaison from Edmonton and surrounding towns, and included two imams, one executive director, two board members, and one mosque operations manager.

**Table 1:** Characteristics of participants

Characteristics	*n*	*%*
Gender		
Female	46	(52.2)
Male	42	(47.7)
Age (years)		
55 and under	21	(23.8)
56–65	31	(35.2)
66–75	27	(30.6)
76–85	9	(10.2)
Place of birth		
Ethiopia	13	(14.7)
Lebanon	25	(28.4)
Somalia	17	(19.3)
Other	33	(37.5)
Immigration status (*n* = 84)		
Economic immigrant	16	(19)
Sponsored by a family	38	(45.2)
Refugee	19	(22.6)
Super visa/visiting visa	4	(4.7)
Study permit	3	(3.5)
Other	4	(4.7)
Years in Canada		
<5	3	(3.4)
5–10	6	(6.8)
>10	79	(89.7)
Years in Edmonton		
<5	5	(5.6)
5–10	9	(10.2)
>10	74	(84.0)
Highest education		
No formal education background	2	(2.2)
Elementary	17	(19.3)
Secondary	29	(32.9)
Post-secondary	40	(45.4)
Dwelling		
Spouse/partner	28	(31.8)
Spouse/partner and children	16	(18.1)
Children	20	(22.7)
Multi-generation	2	(2.2)
Alone	19	(21.5)
Other	3	(3.4)
Main source of income		
Wages and salaries	29	(32.9)
Income from self-employment	4	(4.5)
Employment insurance	2	(2.2)
Worker’s compensation	3	(3.4)
Benefits from Canada or Quebec Pension	6	(6.8)
Retirement pensions	24	(27.2)
Old-age security	13	(14.7)
Provincial or municipal social assistance or welfare	4	(4.5)
No formal source of income	7	(7.9)
Most-often-used language		
Arabic	34	(38.6)
English	22	(25)
Somali	20	(22.7)
Harar	9	(10.2)
Other	8	(9)
Other used language		
Arabic	42	(47.7)
Amharic	11	(12.5)
Urdu	11	(12.5)
Somali	9	(10.2)
Other	21	(23.8)

### Key findings

Three interconnecting themes were identified from the survey results and qualitative interviews: (1) triple jeopardy leading to loneliness, (2) decreased access to resources for connectivity, and (3) organizational struggles to provide support during the pandemic (Table 2). The socio-ecological model was used to categorize the pandemic-related factors identified across themes that influenced the well-being of Muslim immigrant older adults (Fig. 1).

**Table 2:** Derivation of themes from group of codes and quote exemplars

Themes	Group of codes	Related quote exemplars
(1) Triple jeopardy leading to loneliness	Fear of COVID, lack of physical encounter with people (public health restrictions), racism and/or violence, no impact	“No one can visit me. 6–7 months I did not see anyone. It was really difficult” (P4) “Even if children come to check in, the way we used to interact has changed.” (P1)
(2) Decreased accessibility to resources for connectivity	Digital technology, lack of spiritual/religious activity (mosque), transportation, family, and social network	“I told him that I feel suffocated. I want to see people, meet people and there is no place for me other than the mosque…I went with him.” (P9) “Seniors often needed technological support to join and attend online events by the mosque or to connect with people.” (Stakeholder 3)
(3) Organizational struggles to provide support during the pandemic	Mosque offered support and programs, funding for organizations, central role of mosques in Muslim communities	“Provided meals to needy families/seniors.” (Stakeholder 4) “Designated funding from the government for Muslims (marginalized) populations required.” (Stakeholder 5)

#### Triple jeopardy leading to loneliness

Most respondents reported the same mental health status before and during the pandemic (57.95%, 51/88). A total of 32 (36.36%) respondents reported worse mental health during the pandemic (somewhat worse: 30.68%, 27/88; much worse: 5.68%, 5/88). For physical health status, 48 (54.55%) of the respondents reported no change during the pandemic; however, 38 (43.19%) respondents reported worse physical health status after the pandemic started (somewhat worse: 29.55%, 26/88; much worse: 13.64%, 12/88). Loneliness was a common experience; 39 (44.32%) of the respondents reporting feeling lonely during the pandemic (Table 3) citing reasons as lack of contact with family, lack of community connectivity via local mosques, and social exclusion: “Not being able to meet children and grandchildren, not being able to socialize normally gave rise to feelings of loneliness and isolation. The ‘human contact’ was missing” (Participant 01). Stakeholder (04) confirmed this experience: “They (older adults) yearn for social interaction and the lack of it has caused loneliness and isolation.” Many participants also reported fear as a key factor. Older adults were afraid to go out and contract the virus, and family members were afraid of unintentionally transmitting the virus to older adults.

Even if our children came to check in, the way we used to interact had changed. To not be able to hug each other in fear of spreading the virus was heart-breaking. It was more difficult for people who were dependent on others as they couldn’t socialize or go out on their own. (Participant 01)

**Table 3:** Participants’ responses on their mental and physical health status, the frequency of feeling lonely, and missing supports during the pandemic (*n* = 88)

Responses	*n*	*%*
Mental health		
Much better now	1	(1.1)
Somewhat better now	4	(4.5)
About the same	51	(57.9)
Somewhat worse now	27	(30.6)
Much worse now	5	(5.6)
Physical health		
Much better now	1	(1.1)
Somewhat better now	1	(1.1)
About the same	48	(54.5)
Somewhat worse now	26	(29.5)
Much worse now	12	(13.6)
Feeling lonely		
Never	49	(55.6)
Sometimes	15	(17)
Often	13	(14.7)
Always	11	(12.5)
Missing supports		
Transportation	7	(7.9)
Nutritious food	2	(2.2)
Affordable housing	10	(11.3)
Housekeeping/home maintenance	4	(4.5)
Medical/health services	3	(3.4)
Grocery shopping	5	(5.6)
Social/recreation opportunities	19	(21.5)
Had all the needed supports	54	(61.3)

Mosques and ethnocultural organizations allow older adults to connect with people with shared cultural, historical, and religious practices. The communal aspect of prayer and religious activities promotes connectivity and community solidarity; however, this was lost during the pandemic.

It was a very tough time for the entire Muslim community for not being able to attend mosque in person. Coming to the mosque to pray and socialize is an integral part of life for Muslims, especially the seniors in the community. It was like a ‘second home’ to them. (Stakeholder 03)

Another stakeholder highlighted the impact on older women with limited opportunities for social engagement:

It has been particularly difficult for women in the community as engaging in mosque-related activities was their only way to get out of their home and engage with others. Not being able to go out and relieve the tension has led to increased conflicts within families. (Stakeholder 04)

The fear of COVID-19 transmission coupled with the fear of racism encountered by some older adults, especially those from Black Muslim communities, amplified the negative impacts of the pandemic. Incidents of violence driven by racism and fewer people in public spaces increased fear of accessing essential services: “Black Muslims have encountered several kinds of barriers and racism in the system during the pandemic putting them at more risk of isolation” (Stakeholder 05). A newcomer older adult not fluent in English faced extreme loneliness, representing a specific group of older adults who experienced heightened vulnerability: “Difficulty moving around due to fear of using bus, so lack of transportation. Lack of access to groceries as cannot order online and not comfortable going out. No one can visit me” (Participant 04).

For some participants, the pandemic did not impact social isolation and loneliness because they had strong family networks, social support, and personal resources. However, those who did experience loneliness and isolation reported that fear, racism, and decreased social connectivity heightened their vulnerability.

#### Decreased access to resources for connectivity

As the pandemic progressed, virtual social and religious programming was introduced by the mosque to enhance connectivity. The majority of survey respondents were interested in attending in-person programs (93.18%, 82/88), especially for religious studies, social and recreation activities, and exercise (56.82%, 50/88; 50%, 44/88; 45.45%, 40/88). Most respondents reported no barriers to attending these programs before the pandemic, but 29 respondents reported barriers with mobility, transportation, lack of time, health issues, and language. Respondents had less interest in attending virtual programs during the pandemic than in-person programs, but almost all (86) participants reported some interest in attending online programs: 52 (60.47%) respondents reported “would like to attend” or “may attend” and 30 (34.88%) respondents reported “not willing to attend.” The most preferred virtual programs were religious study, social and recreation activities, and exercise (40.91%, 36/88; 32.85%, 29/88; 28.41%, 25/88). A majority of participants reported no need for technology support nor being contacted to receive support, while 28 (32.18%) participants reported that they needed technical assistance, a point emphasized by stakeholders: “Some senior members experience difficulty with technology (do not have know-how of technology) to register for programs by the mosque which leads to the inability to engage in programs” (Stakeholder 04) and “Seniors need support from the younger generation regarding matters like technology, registering for programs, and even daily tasks” (Stakeholder 06).

Table 3 presents survey responses regarding missing supports during the pandemic. Along with technological difficulties, lack of transportation was a barrier to accessing resources both before and during the pandemic. Before the pandemic, older adults could ride-share with other members of the community or access other ride services, but pandemic restrictions prevented participants from commuting for basic needs like groceries, a doctor’s visit, or attending the mosque. Many stakeholders emphasized the need for more accessible and dedicated transportation services for older adults to facilitate connectivity: “It was particularly difficult for seniors who were dependent on others as they were not able to commute to mosque or social groups and transportation was a huge problem for them.”

In contrast, digitally literate older adults used technology to connect with family, friends, and religious communities:

To be able to use technology to connect with peers, family, senior groups or with the events around the world helped with the ‘social’ aspects of life. The senior group from the mosque hosted activities regularly on Zoom like chair yoga which gave a chance to socialize and interact with other senior members. It was beneficial for seniors suffering from loneliness as it gave them an opportunity to express their feelings and to learn something too. (Participant 2)

The mosque offered very ‘successful and strong’ online programs for the Muslim community like Salah (prayer) and Sermons. These programs were especially fruitful for the seniors in the community as attending mosque was a very important activity in their life which was disrupted. (Stakeholder 03)

Digital literacy hindered participation in virtual programming at the mosque, while transportation barriers existed before and during the pandemic. Importantly, respondents indicated a lack of interest or ability to participate in virtual programming during the pandemic.

#### Organizational struggles to provide support during the pandemic

The pandemic also affected how the organizations continued to provide support to their communities during lockdowns with reduced funding and resources:

The mosque suffered financially as there were fewer people (due to restrictions) to contribute funds to the mosque. Financial support was needed for the mosque during the pandemic to run its daily expenses. (Stakeholder 02)

The number of Muslims approaching mosques for guidance and help with family matters increased significantly during the pandemic. Stakeholders reported receiving an immense number of calls for help regarding domestic violence, mental health struggles, and food insecurity.

There have been increased cases of family conflicts, abuse, and divorces in the community. There are limited resources within the Muslim communities to address issues like abuse, divorces, and mental health. (Stakeholder 04)

The pandemic greatly impacted the economic and financial situation of many households. Unemployment, salary cutbacks, and lay-offs forced people to seek support from their local organizations, who reported an increased number of people approaching them. The executive director of a prominent organization recalls:

The number of people approaching the organization for accessing necessities like food increased significantly over the pandemic. The demand for resources (foodbank, groceries) increased and the funding was limited…All the funds had to be diverted to purchasing goods (groceries) for the people approaching the organization due to increased demand. (Stakeholder 05)

Another stakeholder mentioned that not all foodbanks were culturally sensitive, and Muslim community members who used them would often not be able to consume most of the items offered to them. “The pandemic led to increased use of foodbanks by families but those are not culturally sensitive (halal)” (Stakeholder 04).

Overall, the capacity of mosques and other local ethnocultural organizations to support Muslim communities during the pandemic was stretched with an increased number of requests for support and exacerbated by lack of funding.

## Discussion

This study explored the experiences and needs of Muslim older adults during the COVID-19 pandemic. Pandemic experiences and related needs of this population were diverse and contingent on several factors, and we draw on the socio-ecological model to discuss some key factors below.

### Individual determinants of digital literacy

Canadians 65 years of age or older and those with underlying health conditions are particularly susceptible to adverse outcomes, including death, from COVID-19 infections (Government of Canada, 2019; Wang et al., 2021). The pandemic led to further marginalization of older adults and normalized ageism in public discourse (D’Cruz & Banerjee, 2020; Wang et al., 2021). While the negative consequences of the pandemic on older adults are widely discussed here and elsewhere, another study reported that many older adults were resilient during the pandemic (Fuller & Huseth-Zosel, 2020). We found that digital literacy was an important resource for Muslim older adults, allowing them to access information, communication, and entertainment during isolation and lockdowns (Beaunoyer et al., 2020). Many study participants reported that digital competence was an asset during these times, and digital technologies can be a tool to counter loneliness and improve the well-being of older adults. However, digital inequities for older adults persist (Martins Van Jaarsveld, 2020). While some participants were interested in digital programming and enhancing digital literacy skills, others were not. Motivating factors for digital adoption and ways to support digital learning in this population should be further explored.

### Social determinants of family and mosque supports

Social determinants constitute the social structures around individuals that promote social cohesion and connectedness (Klasa et al., 2021). Family relationships are central to immigrant older adults and provide needed resources, solidify cultural identity and belonging, and enhance resilience (Nasir et al., 2022). Relationships to social groups and local community organizations are also important (Ishikawa, 2020). Older adults living alone with no social or family interactions were severely impacted by physical distancing requirements, increasing the risk of mental health problems like anxiety and depression (Garcia et al., 2020). Additionally, being confined at home for long periods with no opportunity to socialize led to family conflict and increased risk of abuse toward elders or women (Ekwonye et al., 2021; Mesa Vieira et al., 2020). Stakeholders, but not older adults, reported an increase in family conflict during the pandemic. Exploring family conflict and the impact on Muslim and racialized immigrant communities should continue because of the ongoing economic and social repercussions of the pandemic. In addition, the role of religion in buffering or coping with stressors should be explored.

The experience of the pandemic was more challenging for racialized community members, especially Black Muslim older adults, with racism cited as a main contributing factor. Black immigrant communities were disproportionately affected during the pandemic because of living and working conditions that increased exposure to the virus, lack of access to healthcare resources, and discrimination (Garcia et al., 2020; Hearst et al., 2021). A more comprehensive study is needed of the lived experiences of older adults simultaneously experiencing race, age, and gender-based exclusion in Canada during public health crises. 

### Natural and human-moderated pandemic environment

The COVID-19 virus re-shaped the natural environment of older adults: contaminated air and surfaces became a source of fear and anxiety, which drastically changed the patterns of daily life (Hearst et al., 2021). Public health restrictions and policies shut down many essential services for extended periods of time. Although these measures were essential, barriers to accessing necessities were exacerbated for certain populations (D’cruz & Banerjee, 2020; Hearst et al., 2021; Nöstlinger et al., 2022). Older adults and stakeholders reported decreased social connectivity from lack of access to transportation before and during the pandemic and closure of mosques during the pandemic. Fear of infection while using public transportation and the inability of family and friends to drive older adults to the mosque were specific to the pandemic. Many community-based initiatives and organizations increased supports for their more vulnerable community members (Nöstlinger et al., 2022), but mosque stakeholders reported that mosques had limited ability to provide support because of limited funding and volunteers.

Although Muslim older adults faced the same aging-associated challenges during the pandemic (i.e., social isolation, loneliness) as their non-immigrant non-Muslim counterparts, the experiences of the former were intensified because of social, emotional, and economic exclusion (Salma & Salami, 2019). Immigrant older adults often experienced racism and discriminatory behaviour (Islamophobia), limiting their ability to be present in public spaces and alleviate their isolation and loneliness. Social isolation, loneliness, and exclusionary practices (with the added burden of the pandemic) separate older immigrants from mainstream society, and policy makers and researchers need to adopt an intersectional and exclusionary lens to find ways to integrate older immigrants into society. A systems approach to identifying and modifying systemic problems creating vulnerabilities among this population is also important.

### Strengths and limitations

Conducting CBPR during a pandemic with older adults is challenging; however, in addition to obtaining rich data, the surveys and interviews can be “care work” (Averett, 2021; Liegghio & Caragata, 2020) and provide an opportunity to connect with isolated older adults in the community. It was difficult to reach the older-age cohort, so this study had a younger sample of older adults (young-old). Ways to connect with the oldest-old during times of crises should be explored, as this group might be more isolated and lack access or familiarity to digital connections. Caregivers and family members could be used as proxy informants for older adults, especially in family-centric communities where multi-generational living is common. Our results are also not generalizable beyond the Muslim community in Edmonton because we used a small sample of a localized population of mainly long-term immigrants (living in Canada for greater than 10 years) who were part of an existing mosque congregation. Our recruitment approaches (phone and in-person information booth) meant we only could access older adult respondents who were already engaged in the mosque. Additionally, the active working status of participants was not directly assessed, which could have indicated the impact of work on stress and isolation. Finally, we only collected survey data on self-rated changes in mental health, loneliness, and isolation at one time point during the pandemic. Future research should assess these factors during the later pandemic stages and post-pandemic to estimate the impact of the pandemic and the development of resilience in older adults during the pandemic. However, this study is a rich case exemplar because our sample had equal gender distribution and well-distributed diversity across common immigration, language/ethnicity, living arrangements, and education categories. The inclusion of “check-in” surveys and rapid qualitative methods allowed the CBPR partners to quickly identify community needs, and this approach could be used during future crises.

## Conclusion

There is limited research on the experiences and needs of immigrant older adults during the COVID-19 pandemic. A variety of studies are being published regarding the vulnerability of older adults during the pandemic, but further research is required on immigrants who face additional barriers and challenges. This study highlights the various impacts of the pandemic on immigrant older adults and the community-based organizations supporting them and emphasizes the role of faith-based and ethnocultural organizations to cater to their communities’ needs during times of crisis.

## Contributions to knowledge

What does this study add to existing knowledge?

- Lack of access to mosque-based resources negatively impacted the well-being of older adults during the pandemic.
- Low digital literacy, transportation barriers, closure of religious centres, and racism heightened loneliness and isolation in Muslim older adults.
- Mosques were sites of support for older adults in Muslim communities but lacked capacity to provide needed supports.

What are the key implications for public health interventions, practice, or policy?

- This is a case example of the experiences of Muslim older adults in an urban Canadian city and offers insights into the importance of mosque-based supports.
- The study highlights the relevance of digital literacy, family support, transportation, and racism to experiences of social isolation and loneliness during the pandemic.
- Interventions targeting digital literacy could be explored further with this population.

## Supplementary Information

Below is the link to the electronic supplementary material.

Supplementary file1 (DOCX 15 KB)

## Data Availability

Not applicable.
