# Supplementary material for: Impact of the COVID-19 pandemic on Muslim older immigrants in Edmonton, Alberta: A community-based participatory research project with a local mosque
Source: Can J Public Health. 2023 Mar 30;114(3):378–88. doi: 10.17269/s41997-023-00764-7 (PMC10062246; doi:10.17269/s41997-023-00764-7)
Supplement: Supplementary file 1 — Supplementary file1 (DOCX 15 KB) [file 41997_2023_764_MOESM1_ESM.docx]

*Interview Guide*

| Sample Questions-Older Adults | Sample Questions-Stakeholders |
| --- | --- |
| 1. How has life been for you over the last year during the pandemic? 2. What are some challenges you experienced, if any? What are some strengths you see in yourself that allowed you to cope positively during the pandemic? 3. Did your social connections change from before to now during the pandemic? 4. What role has technology played in staying connected during the pandemic? 5. Now that there are vaccines for COVID-19, did you or are you planning to be vaccinated? What are your thoughts about the vaccine? 6. The Mosque wants to start some programming for seniors, what types of programs would you like to see? 7. Are there barriers that would prevent you from attending the mosque in person? From attending virtual programs? | 1. Are there many Muslim seniors in your community and do they attend the mosque? 2. If seniors are not attending the mosque, can you explain why this is? 3. How has the pandemic impacted Muslim seniors? 4. Have you noticed more social isolation or loneliness in Muslim seniors during the pandemic? 5. What are some supports that Muslim older adult need in the community? 6. What types of programs and supports for seniors would you like to see at your Mosque? 7. We are creating a report that will go to government and other decision-makers, is there anything relevant to the needs of the Muslim community during the pandemic that you would like us to include in this report? |
